# Metal Nanoparticles–Polymers Hybrid Materials II

**DOI:** 10.3390/polym14091901

**Published:** 2022-05-06

**Authors:** Iole Venditti

**Affiliations:** Sciences Department, Roma Tre University, Via della Vasca Navale 79, 00146 Rome, Italy; iole.venditti@uniroma3.it; Tel.: +39-06-5733-3388

Despite the pandemic, the last five years have been marked by an extraordinary development of new advanced technologies, based not only on new materials but also on modeling, information technology and artificial intelligence, which have allowed for great leaps forward in various research fields [1,2,3,4]. This Special Issue, which follows the success of a previous edition on the same topic, fits into this context. In fact, these advanced materials combine the advantages of the different components that often have synergistic properties and effects [5,6,7,8,9]. Naturally, this has opened a very active front of study and research, which provides substrates and ideas regarding industrial needs and applications, ranging from medicine, to biotechnology, optoelectronics, sensors and energy [10,11,12,13,14,15,16]. This Special Issue focuses on highlighting the progress in new hybrid materials, based on metal nanoparticles and polymers, as well as their preparation, functionalization, characterization, and smart application in advanced fields.

Nabil Bouazizi et al. [17], created a review of the amine-surface functionalization of inorganic adsorbents for water treatment. Various inorganic adsorbents (INAD) were presented, based on metals, metal oxide, graphene oxide, and metal–organic structures, functionalized by grafting amines to obtain the removal of pollutants in wastewater. In fact, the INAD functionalized with amines was successfully used to adsorb and remove heavy metals, dyes, organic molecules, mixtures of pollutants and bacteria from the water. In addition, amine-modified INADs enhance antibacterial activity due to the presence of highly reactive oxygen species. The review presented several studies in which surface functionalization with amines can occur through covalent interactions, hydrogen bonds and electrostatic interactions, depending on various experimental parameters, such as solvent, temperature, amount of water and amino precursors. In coming years, the challenge will be to scale these laboratory procedures down to the industrial production level.

A. B. Shcherbakov et al. [18] present a review summarizing the state of the art and perspectives for the use of cerium oxide nanoparticles in advanced polymer composites for biomedical applications. Nanoceria is a known as an inorganic mitogen and stimulates cell proliferation, accelerating wound healing in vivo. Nanoceria reduces inflammation and the autoimmune response, and possesses antimicrobial and anti-biofilm properties. The studies included in this review demonstrate that the polymer composites containing nanocrystalline combine the advantages that are exhibited by each component of the material and reduce the disadvantages inherent in both the nanocrystalline and polymer matrices. A major focus of this review is the positive effect of nanoceria on advanced biomedical polymeric materials, such as its UV protection properties, biomolecule and cell adhesion, biocompatibility, etc. Therefore, the composites allow for the advantages exhibited by each component to be combined, increasing the potential and efficacy of the biomedical applications of both nanoceria and polymer matrices.

S. Zhang et al. [19] proposed a controllable synthesis of polystyrene (PS) spheres by emulsion polymerization, emulsion polymerization without emulsifiers (EFEP), by adjusting the polymerization reaction time, the ionic strength and the concentration of the ionic copolymer. A simple layer-by-layer assembly (LBL) method was employed to fabricate multi-layer 2D PS/Ag Surface Enhanced Raman Scattering (SERS) substrates with a controllable enhancement effect. The 10-layer PS/Ag 2D substrate showed the best SERS improvement, which was almost twice as strong as the use of colloidal PS/Ag alone. More importantly, the PS/Ag 2D substrate assembled from LBL showed excellent reproducibility. The LBL PS/Ag substrate exhibits high activity for SERS, which can detect harmful pesticides, i.e., malachite green (MG) and dimetridazole (DMZ) in food samples, with a limit of detection as low as 3.5 ppb. This 2D SERS substrate, using plasmonic PS and NP spheres, can be applied as an effective biosensor to detect other types of pesticide residues in food samples to improve food quality.

Md. K. Haider et al. [20] prepared an antibacterial electrospun polyacrylonitrile (PAN) nanofiber membrane, which was surface-coated with lignin-synthesized silver nanoparticles (AgNPs). Scanning electron microscope (SEM) observations show a smooth, beadless morphology of the nanofibers, with a slight increase in the average diameters of the nanofibers. Transmission electron microscope (TEM) images and inductively coupled plasma-atomic emission spectrometry (ICP-AES) revealed that the amount of AgNPs formed is proportional to both the precursor salt and the alkaline lignin concentration. X-ray photoelectron spectroscopy (XPS) analyses confirm the presence of AgNPs on the surface of the nanofibers. In addition to synthesizing AgNPs, the lignin layer observed on the surface of the nanofibers showed a multifunctional performance at PAN/AgNPs. The hydrophobic PAN nanofibers showed hydrophilicity and good liquid absorption properties after coating. The free radical scavenging ability was observed for all PAN/AgNPs resulting from the surface-coated lignin. The resulting composite nanofibers showed excellent antibacterial activity against Gram-positive *S. aureus* and Gram-negative *E. coli* bacterial strains.

Gold nanohybrids conjugated with folate-hydrophobic-quaternized pullulan delivering hydrophobic camptothecin (CPT-GNHs@FHQ-PUL) were prepared by S. Laksee et al. [21] as a promising multifunctional nanocarrier, capable of delivering CPT, a hydrophobic anticancer drug, in human lung cancer cells (Chago-k1). The hybrid system has the characteristic localized surface plasmon resonance (LSPR) band at 528 nm and an average size of approximately 10.97 ± 2.29 nm. Smart GNHs@FHQ-PUL is CPT compatible, forming self-assembly via intermolecular interactions. The study of the anticancer activities of CPT-GNHs @ FHQ-PUL showed an improvement in CPT activity of about 2.82 times, with a consequent reduction in the IC50 values compared to CPT alone. CPT-GNHs @ FHQ-PUL were absorbed into cells by folate receptor-mediated endocytosis, hydrophobic interactions, and electrostatic interactions. TEM and confocal laser-scanning microscope (CLSM) images revealed that CPT-GNHs @ FHQ-PUL were dispersed in the cytoplasm, accumulating around the nucleus. The release behaviors of the CPT-GNHs @ FHQ-PUL nanohybrids were pH-dependent, offering a faster CPT release from these systems at pH 5.0, compared to pH 7.4. The results of this study showed that the delivery of CPT by smart GNHs@FHQ-PUL systems proved to be a promising strategy to increase its chemotherapeutic effects.

K. Bukovinszky et al. [22], reported a study in which AuNPs, of 5 nm size and functionalized with dodecanethyol, are embedded in dental methacrylate resin containing Irgacure 784 photoinitiator. Measurements of the change in refractive index and Raman microscopy/confocal Ramaer spectroscopy made it possible to determine the degree of conversion and to monitor the course of the polymerization reaction in the resin. In this study, therefore, a new experimental resin was produced based on methacrylate, which has better physical and chemical properties than the reference resin. The work is also of clinical importance, as this hybrid can be applied as a resin matrix of an experimental dental composite.

D. Toyen et al. [23], determined the thermal neutron and self-emitted gamma attenuations of ultra-high-molecular-weight polyethylene (UHMWPE) composites containing varying Sm_2_O_3_ contents of 0–50 wt.% at a neutron energy of 0.025 eV and varying gamma energies of 0.334, 0.712, and 0.737 MeV, using a simulation code, namely, MCNP-PHITS. The current work also investigated the effects of a silane-coupling agent on the morphological, wear, frictional, and mechanical properties of UHMWPE composites containing 25 wt.% of Sm_2_O_3_. The results from the PHITS simulation indicated that the ability to attenuate thermal neutrons and self-emitted gamma rays of the composites increased with the increasing Sm_2_O_3_ contents, while the gamma-shielding properties of the composites tended to decrease with increasing gamma energies. The comparison of the thermal neutron-shielding properties of the Sm_2_O_3_/UHMWPE composites with a commercial material containing 5% boron indicated that the composites with Sm_2_O_3_ contents of 11–13 wt.% could attenuate thermal neutrons with equal efficiency to the commercial product. The overall outcomes of this work suggested the great potential of utilizing Sm_2_O_3_ as a superior neutron attenuator in UHMWPE composites, with a preferred dual-shielding property. Furthermore, the surface treatment of Sm_2_O_3_ with 5–10 pph KBE903 could successfully enhance the wear resistance and strength of the composites, increasing the usability and processability of the product.

S.-R. Son et al. [24], fabricated a functional hybrid polyimide (PI) alignment layer that reduces the difference in refractive index and greatly increases the transmittance of the device by introducing inorganic titanium dioxide (TiO_2_) nanoparticles (NP) to the organic PI. The TiO_2_ nanoparticles of 15 and 300 nm in size were surface-treated with stearic acid, and the PI mixture with TiO_2_ NPs was spin-coated on an ITO substrate and then imidized to form a hybrid alignment layer. The interactions between the long hydrophobic alkyl chains of stearic acid on the surface of the TiO_2_ NPs and the liquid crystal (LC) molecules was expected to improve the vertical alignment ability of the LC molecules, thereby improving the electro-optical properties of the device. In addition, the dark state observed under cross-polarization indicated a complete vertical alignment of the LC device with hybrid PI layers, and the relatively high threshold voltage and fast decay response time of the device suggested the improved vertical alignment ability of the hybrid PI layer. Optical transmittance of LC cell was enhanced by incorporating TiO_2_ NPs into the PI layer, and the transmittance increased as the size of the TiO_2_ NPs increased. The long-term driving evaluation indicated that the functional hybrid PI layer with TiO_2_ NPs improved the reliability of the device. From the perspective of the alignment material, the proposed method using a hybridization of organic PI and surface-treated TiO_2_ NPs is expected to have a significant impact on the display field because it allows for a simple preparation process, stable vertical orientation of LCs and high optical transmittance, and ensures the long-term reliability of the device.

Anna Słubik et al. [25] showed that the iron(III) oxide nanoparticles can be used as a crosslinking agent for the elastomeric blends containing chloroprene rubber (CR) and butadiene rubber (BR). Additionally, the use of nano-iron(III) oxide to crosslink the CR/BR blends enables the controlled conduct of interelastomeric reactions. The presence of additional bonds between the rubbers used helps to obtain vulcanizates characterized by their good mechanical properties and very high resistance to fire. The results of the conducted research show that chalcedony or sillitin can be used as alternative fillers in the elastomer technology. The dynamic tests exhibit that the occurrence of filler–filler or filler–rubber interactions depends on the type of filler used. Ultrasil can create its own structure in the elastomeric matrix and, in the case of a sample containing aerosol, it is possible to create filler–rubber interactions. The incorporation of sillitin or chalcedony only leads to an increase in the storage modulus. The possibility of creating filler–filler or filler–rubber interactions in the elastomeric matrix affects the functional properties of the vulcanizates that are produced. The CR/BR blend filled with ultrasil was characterized by the highest increase in the torque after 30 min of heating. The presence of sillitin or chalcedony contributes to the production of vulcanizates with a similar degree of cross-linking. In the case of mechanical properties, it was observed that the presence of aerosil significantly increased the tensile strength (TSb = 19.80 MPa). The incorporation of the fillers enables the production of fire-resistant rubber materials, as evidenced by the high oxygen index (OI > 37.5%). Unfortunately, these vulcanizates show poor resistance to aging factors, but after the incorporation of an antiaging substance, compositions designed in this way can be used for the production of many rubber materials. Their increased resistance to fire allows them to be used in the production of protective clothing, fire seals, or conveyor belts.

G. Buema et al. [26] synthesized hybrid inorganic CoFe_2_O_4_/carboxymethyl cellulose (CMC) polymeric framework nanobead-type adsorbents with tailored magnetic properties. The prepared material was characterized by means of SEM, powder X-ray diffraction analysis (XRD), thermogravimetric analysis (TG), and vibrational sample magnetometry (VSM). A precise self-assembly engineering of their shape and composition, combined with an in-depth testing for cadmium removal from wastewater, was provided. The adsorption performance of CF@CMC was evaluated using Cd (II) in an aqueous solution. Initial metal concentration, contact time and adsorbed dosage were the parameters studied for the adsorption experiments performed in this study. The adsorption process is dependent on the adsorbent dosage, initial cadmium concentration, and contact time. The results show that the optimum value of adsorbent dosage is 1.4 g/L. Capacity uptake increases with the increase in initial concentration, reaching a plateau at a maximum of 150 mg/L. The Langmuir adsorption isotherm model and pseudo-second-order model describe the adsorption uptake of the Cd (II) from aqueous solutions using the magnetic nanobeads. The employed models were fitted best with a maximum capacity uptake of 44.05 mg/g. The synthesized adsorbent has the benefits of being cheap and easily obtainable. Generally, the results show that this type of material can be applied as a potential adsorbent for the removal of cadmium ions from aqueous solutions.

In conclusion, as editor of this Special Issue, I am aware that the diversity and innovation of the new compounds and tools that are rapidly developing in multidisciplinary research related to nanomaterials based on noble metals cannot all be collected in a single volume. However, I am sure that this collection will increase the research interest in this area, providing our readers with a broad and updated overview of this topic.
